# Supplementary material for: Effects of 12-Month Training Intervention on Physical Fitness, Body Composition, and Health Markers in Finnish Navy Soldiers
Source: Healthcare (Basel). 2023 Oct 9;11(19):2698. doi: 10.3390/healthcare11192698 (PMC10572769; doi:10.3390/healthcare11192698)
Supplement: Supplementary file 1 [file healthcare-11-02698-s001.zip › healthcare-2624175-supplementary.pdf]

12-minute running test	
**Citeria:** Improvement in the test calclulated by meters per person	
**Position of the Team**	**Scoring**
1. position	8 points
2. position	6 points
3. position	5 points
4. position	4 points

Other primary physical fitness tests (push-ups and sit-ups in one minute, standing long jump)	
**Citeria:** Improvement in the tests calclulated in percentage per person	
**Position of the Team**	**Scoring**
1. position	7 points
2. position	5 points
3. position	4 points
4. position	3 points

Conduction of the training program and other exercises	
**Citeria:** Conduction of the training program and other exercises in percentage per person	
**Position of the Team**	**Scoring**
1. position	6 points
2. position	4.5 points
3. position	3.5 points
4. position	1.5 points

Total score of the team

Figure S1. The scoring of the teams. The teams scored points for the changes in the 12-minute running test and other primary physical fitness tests between the baseline- (PRE) and end-measurements (POST). Points were also scored for completion of the training program and other exercises. Two teams that scored the most points were rewarded after the intervention. Completing the training program and other exercises was evaluated as the percentage per person. Performing one primary training session in a week counted for 35% of the week's total and 1 secondary training session was 15% of the week's total. When completing the whole training program during a week (2 primary and 2 secondary training sessions), it was possible to achieve 100%. During the service at sea, two primary strength training sessions, that were designed especially for the naval environment, were enough to achieve 100% in a week, because of the limited possibility to exercise on duty. To motivate the participants for other free time sports, other reported exercises (not included in the training program) were evaluated as 7.5%/1hour. In the intervention T4 scored the most points (18p), T3 was second (17.5p), T1 was third (13.5p) and T2 was fourth (8.5p).

Strength / Endurance / Combined training	Type of training	Training session	More detailed content of the training sessions
Strength training	Circuit training	Strength 1	10 different moves, 30s exercise time per move, 30s pause between moves, two circuits
		Strength 2	
	Muscle endurance training	Strength 3	40–60% of 1RM, 10–12 reps
	Optimal training for muscle hypertrophy	Strength 6	60–80% of 1RM, 6–8 reps
		Strength 9	50–75% of 1RM, 8–10 reps;
		Strength 10	60–80% of 1RM, 6–8 reps
	Maximal strength training	Strength 4	75–90% of 1RM, 3–5 reps
		Strength 5	
		Strength 7	
		Strength 8	
Endurance training	Basic endurance training	Endurance 1	Heart rate < 140 bpm
		Endurance 5	
		Endurance 7	
		Endurance 8	
	Low intensity interval training	Endurance 2	Heart rate 140–160 bpm
	High intensity interval training	Endurance 3	Heart rate > 160 bpm
		Endurance 4	
		Endurance 6	
Combined strength and endurance training	Muscle endurance and basic endurance training	Combined 1	40–60% of 1RM, 10 reps and heart rate < 140 bpm
		Combined 2	
		Combined 3	
		Combined 5	
	Optimal training for muscle hypertrophy and basic endurance training	Combined 4	60–80% of 1RM, 6–8 reps and heart rate < 140 bpm
	Explosive strength and high intensity interval training	Combined 6	30–50% of 1RM, 3–5 reps and heart rate > 160 bpm

Figure S2. The training sessions of the training program. All training sessions started with a warmup and ended with mobility exercises. During service at sea it was not possible to follow the training program and the participants were advised to perform additional strength training sessions that were especially designed for the naval environment. The strength training during service at sea contained six different training sessions. The environment on a missile patrol boat is quite isolated and confined, but pull-ups, push-ups, sit-ups, squats, and exercises with kettlebells could be performed on the deck of the vessel, while the boat was visiting harbor. Three of these training sessions were total body exercises, one was for only the upper body, one for the abdominal muscles/lower back and another for the lower body. All the strength training performed on the vessel contained muscle endurance training, while it was problematic to train with heavy weights on board.

Weeks 1 – 2	PRE-measurements at the baseline			
Week 3	[b]Endurance 1	[l]Endurance 2	[c]Strength 1	[h]Endurance 3
Week 4	[h]Endurance 4	[c]Strength 2	[b]Endurance 5	[h]Endurance 6
Week 5	[e]Strength 3	[m]Strength 4	[b]Endurance 7	[m]Strength 5
Week 6	[o]Strength 6	[b]Endurance 8		
Week 7	[eb]Combined 1	[eb]Combined 2	[o]Strength 9	[eb]Combined 3
Week 8	[ob]Combined 4	[o]Strength 10	[eb]Combined 5	[xh]Combined 6
Week 9	[b]Endurance 1	[l]Endurance 2	[c]Strength 1	[h]Endurance 3
Week 10	[h]Endurance 4	[c]Strength 2		
Week 11	[e]Strength 3	[m]Strength 4	[b]Endurance 7	[m]Strength 5
Week 12	[o]Strength 6	[b]Endurance 8	[m]Strength 7	[m]Strength 8
Week 13	[eb]Combined 1	[eb]Combined 2	[o]Strength 9	[eb]Combined 3
Week 14	[ob]Combined 4	[o]Strength 10		
Week 15	[b]Endurance 1	[l]Endurance 2	[c]Strength 1	[h]Endurance 3
Week 16	[h]Endurance 4	[c]Strength 2	[b]Endurance 5	[h]Endurance 6
Week 17	[e]Strength 3	[m]Strength 4	[b]Endurance 7	[m]Strength 5
Week 18	[o]Strength 6	[b]Endurance 8		
Week 19	[eb]Combined 1	[eb]Combined 2	[o]Strength 9	[eb]Combined 3
Week 20	[ob]Combined 4	[o]Strength 10	[eb]Combined 5	[xh]Combined 6
Week 21	[b]Endurance 1	[l]Endurance 2	[c]Strength 1	[h]Endurance 3
Week 22	[h]Endurance 4	[c]Strength 2		
Week 23	[e]Strength 3	[m]Strength 4	[b]Endurance 7	[m]Strength 5
Week 24	[o]Strength 6	[b]Endurance 8	[m]Strength 7	[m]Strength 8
Week 25	[eb]Combined 1	[eb]Combined 2	[o]Strength 9	[eb]Combined 3
Week 26	[ob]Combined 4	[o]Strength 10		

Weeks 27 – 28	MID-measurements at the middle of the intervention			
Week 29	[b]Endurance 1	[l]Endurance 2	[c]Strength 1	[h]Endurance 3
Week 30	[h]Endurance 4	[c]Strength 2	[b]Endurance 5	[h]Endurance 6
Week 31	[e]Strength 3	[m]Strength 4	[b]Endurance 7	[m]Strength 5
Week 32	[o]Strength 6	[b]Endurance 8		
Week 33	[eb]Combined 1	[eb]Combined 2	[o]Strength 9	[eb]Combined 3
Week 34	[ob]Combined 4	[o]Strength 10	[eb]Combined 5	[xh]Combined 6
Week 35	[b]Endurance 1	[l]Endurance 2	[c]Strength 1	[h]Endurance 3
Week 36	[h]Endurance 4	[c]Strength 2		
Week 37	[e]Strength 3	[m]Strength 4	[b]Endurance 7	[m]Strength 5
Week 38	[o]Strength 6	[b]Endurance 8	[m]Strength 7	[m]Strength 8
Week 39	[eb]Combined 1	[eb]Combined 2	[o]Strength 9	[eb]Combined 3
Week 40	[ob]Combined 4	[o]Strength 10		
Week 41	[b]Endurance 1	[l]Endurance 2	[c]Strength 1	[h]Endurance 3
Week 42	[h]Endurance 4	[c]Strength 2	[b]Endurance 5	[h]Endurance 6
Week 43	[e]Strength 3	[m]Strength 4	[b]Endurance 7	[m]Strength 5
Week 44	[o]Strength 6	[b]Endurance 8		
Week 45	[eb]Combined 1	[eb]Combined 2	[o]Strength 9	[eb]Combined 3
Week 46	[ob]Combined 4	[o]Strength 10	[eb]Combined 5	[xh]Combined 6
Week 47	[b]Endurance 1	[l]Endurance 2	[c]Strength 1	[h]Endurance 3
Week 48	[h]Endurance 4	[c]Strength 2		
Week 49	[e]Strength 3	[m]Strength 4	[b]Endurance 7	[m]Strength 5
Week 50	[o]Strength 6	[b]Endurance 8	[m]Strength 7	[m]Strength 8
Week 51	[eb]Combined 1	[eb]Combined 2	[o]Strength 9	[eb]Combined 3
Week 52	[ob]Combined 4	[o]Strength 10		
Weeks 1 – 2	POST-measurements at the end of the intervention			

Figure S3. The structure of the training program. The primary training sessions are marked in black color and the secondary training sessions in grey color. During the PRE-, MID- and POST-measurements, there were no training sessions to ensure valid results of the physical fitness tests and other measurements. Strength training: [c] = circuit training, [e] = strength endurance, [o] = optimal training for muscle hypertrophy, [m] = maximal strength. Endurance training: [b] = basic endurance, [l] = low intensity interval training, [h] = high intensity interval training. Combined strength and endurance training: [eb] = strength endurance and basic endurance, [ob] = optimal training for muscle hypertrophy and basic endurance, [xh] = explosive strength and high intensity interval training.

Table S1. The results of physical fitness tests. For PRE, MID, POST: the mean and standard deviation (SD) are presented. For the PRE-MID difference and PRE-POST difference: the mean and 95% confidence intervals (CI) are presented. Effect sizes (Cohen's D) and 95% confidence intervals (CI) for IG vs CG difference are shown.

		PRE mean (SD)	MID mean (SD)	POST mean (SD)	PRE-MID mean difference (95% CI)	PRE-POST mean difference (95% CI)	Effect size (95% CI)
Push-ups (reps/min)	CG	41 (9)	-	43 (15)	-	0 (-2 – 2)	
	IG	38 (10)	40 (11)	40 (11)	1 (-1 – 3)	1 (-1 – 3)	0.27 (-0.23 – 0.76)
	T1	35 (12)	40 (11)	40 (12)	1 (-3 – 5)	4 (1 – 7)^	
	T2	42 (11)	43 (13)	39 (15)	-1 (-6 – 4)	-3 (-9 – 2)	
	T3	40 (7)	41 (11)	42 (10)	0 (-6 – 7)	2 (-2 – 7)	
	T4	36 (9)	38 (9)	38 (8)	3 (-1 – 7)	3 (-2 – 7)	
Sit-ups (reps/min)	CG	43 (9)	-	42 (9)	-	-2 (-3 – 0)	
	IG	41 (9)	42 (8)	41 (10)	1 (-1– 2)	0 (-1 – 2)	0.49 (-0.01 – 0.99)
	T1	38 (11)	38 (10)	36 (12)	-2 (-4 – 1)	-2 (-5 – 2)	
	T2	43 (11)	44 (10)	42 (13)	-1 (-4 – 2)	-1 (-5 – 2)	
	T3	41 (7)	45 (8)	42 (10)	2 (-2 – 6)	1 (-2 – 5)	
	T4	41 (5)	43 (4)	44 (6)	3 (-2 – 7)	3 (1 – 5)^^**	
Standing long jump (m)	CG	2.29 (0.24)	-	2.31 (0.22)	-	-0.01 (-0.03 – 0.02)	
	IG	2.25 (0.27)	2.29 (0.26)	2.25 (0.28)	0.00 (-0.02 – 0.03)	-0.01 (-0.03 – 0.02)	0.00 (-0.49 – 0.50)
	T1	2.20 (0.30)	2.31 (0.30)	2.17 (0.30)	0.01 (-0.05 – 0.08)	0.01 (-0.04 – 0.07)	
	T2	2.32 (0.21)	2.29 (0.22)	2.24 (0.24)	-0.03 (-0.10 – 0.03)	-0.07 (-0.12 – -0.02)^*	
	T3	2.26 (0.24)	2.34 (0.27)	2.28 (0.28)	0.00 (-0.04 – 0.05)	-0.01 (-0.07 – 0.05)	
	T4	2.25 (0.31)	2.25 (0.28)	2.29 (0.31)	0.01 (-0.3 – 0.05)	0.03 (0.00 – 0.05)^	
12-minute run test (m)	CG	2621 (259)	-	'2550 (251)	-	-27 (-112 – 59)	
	IG	2556 (323)	2595 (321)	"2603 (312)	-5 (-65 – 54)	-12 (-56 – 32)	0.12 (-0.52 – 0.76)
	T1	2575 (248)	2701 (305)	2637 (246)	-21 (-163 – 121)	5 (-86 – 96)	
	T2	2527 (366)	2560 (318)	2583 (365)	-1 (-105 – 103)	-76 (-161 – 10)	
	T3	2653 (283)	2744 (340)	2646 (294)	77 (-27 – 181)	-9 (-89 – 72)	
	T4	2489 (388)	2451 (298)	2575 (357)	-85 (-283 – 112)	-19 (-125 – 86)	
MVCupper (kg)	CG	108.8 (30.9)	-	108.3 (31.4)	-	-3 (-7 – 1)	
	IG	101 (20)	104 (19)	103 (21)	-1 (-5 – 4)	-1 (-6 – 3)	0.18 (-0.32 – 0.68)
	T1	93 (13)	93 (9)	91 (12)	-4 (-10 – 2)	-5 (-9 – -2)^^	
	T2	108 (15)	107 (10)	107 (15)	-2 (-8 – 4)	-1 (-9 – 7)	
	T3	110 (31)	113 (32)	114 (32)	- 1 (-8 – 7)	-2 (-8 – 3)	
	T4	97 (14)	101 (18)	100 (18)	3 (-3 – 9)	1 (-4 – 6)	
MVClower (kg)	CG	426.0 (120.7)	-	426.2 (132.0)	-	-23 (-43 – -3)^	
	IG	447 (97)	454 (111)	461 (93)	7 (-18 – 31)	13 (-7 – 32)*	0.67 (0.14 – 1.19)
	T1	404 (40)	391 (75)	431 (68)	1 (-35 – 37))	28 (-8 – 64)	
	T2	465 (63)	470 (32)	458 (77)	0 (-40 – 40)	-7 (-53 – 39)	
	T3	470 (149)	481 (190)	492 (141)	13 (-76 – 103)	30 (-8 – 69)*	
	T4	449 (104)	468 (106)	461 (83)	19 (-34 – 71)	10 (-33 – 54)	
SMBT (m)	CG	6.27 (0.92)	-	6.16 (0.85)	-	-0.22 (-0.38 – -0.07)^^	
	IG	6.47 (0.72)	6.36 (0.86)	6.28 (0.82)	-0.20 (-0.35 – -0.05)^^	-0.23 (-0.41 – -0.04)^	-0.04 (-0.55 – 0.46)
	T1	6.27 (0.62)	6.12 (0.82)	6.10 (0.60)	-0.24 (-0.77 – 0.29)	-0.12 (-0.64 – 0.40)	
	T2	6.56 (0.66)	6.50 (0.68)	6.30 (0.92)	-0.15 (-0.29 – -0.01)^	-0.26 (-0.60 – 0.07)	
	T3	6.87 (0.87)	6.72 (1.26)	6.69 (0.95)	-0.16 (-0.69 – 0.38)	-0.29 (-0.88 – 0.31)	
	T4	6.29 (0.69)	6.19 (0.75)	6.09 (0.74)	-0.14 (-0.42 – 0.15)	-0.20 (-0.49 – 0.09)	

Note. PRE = baseline measurements, MID = measurements in the middle of the study period, POST = measurements after the 12-month study period, PRE-MID difference = difference between the PRE-MID measurements, PRE-POST difference = difference between the PRE-POST measurements, CG = control group, IG = intervention group, T1 = team 1, T2 = team

2, T3 = team 3, T4 = team 4, MVCupper = maximal voluntary contraction of the upper extremities, MVClower = maximal voluntary contraction of the lower extremities, SMBT = seated medicine ball throw. * Significant difference to the control group, $p < 0.05$, ** $p < 0.01$. ^ Significant difference to the baseline value, $p < 0.05$, ^^ $p < 0.01$. ′ means n = 15, " means n = 28.
